# Matrix Conditions and KLF2-Dependent Induction of Heme Oxygenase-1 Modulate Inhibition of HCV Replication by Fluvastatin

**DOI:** 10.1371/journal.pone.0096533

**Published:** 2014-05-06

**Authors:** Andrea Wuestenberg, Janine Kah, Katrin Singethan, Hüseyin Sirma, Amelie Dorothea Keller, Sergio René Perez Rosal, Jörg Schrader, Christine Loscher, Tassilo Volz, Ralf Bartenschlager, Volker Lohmann, Ulrike Protzer, Maura Dandri, Ansgar W. Lohse, Gisa Tiegs, Gabriele Sass

**Affiliations:** 1 Institute of Experimental Immunology and Hepatology, University Medical Center Hamburg-Eppendorf, Hamburg, Germany; 2 Institute of Virology, Technische Universität München/Helmholtz Zentrum Munich, Munich, Germany; 3 Institute of Pathology, University Medical Center Hamburg-Eppendorf, Hamburg, Germany; 4 Department of Medicine I, University Medical Center Hamburg-Eppendorf, Hamburg, Germany; 5 Department of Infectious Diseases, Molecular Virology, University of Heidelberg, Heidelberg, Germany; Bambino Gesu' Children Hospital, Italy

## Abstract

**Background & Aims:**

HMG-CoA-reductase-inhibitors (statins) have been shown to interfere with HCV replication in vitro. We investigated the mechanism, requirements and contribution of heme oxygenase-1(HO-1)-induction by statins to interference with HCV replication.

**Methods:**

HO-1-induction by fluva-, simva-, rosuva-, atorva- or pravastatin was correlated to HCV replication, using non-infectious replicon systems as well as the infectious cell culture system. The mechanism of HO-1-induction by statins as well as its relevance for interference with HCV replication was investigated using transient or permanent knockdown cell lines. Polyacrylamide(PAA) gels of different density degrees or the Rho-kinase-inhibitor Hydroxyfasudil were used in order to mimic matrix conditions corresponding to normal versus fibrotic liver tissue.

**Results:**

All statins used, except pravastatin, decreased HCV replication and induced HO-1 expression, as well as interferon response *in vitro*. HO-1-induction was mediated by reduction of Bach1 expression and induction of the Nuclear factor (erythroid-derived 2)-like 2 (NRF2) cofactor Krueppel-like factor 2 (KLF2). Knockdown of KLF2 or HO-1 abrogated effects of statins on HCV replication. HO-1-induction and anti-viral effects of statins were more pronounced under cell culture conditions mimicking advanced stages of liver disease.

**Conclusions:**

Statin-mediated effects on HCV replication seem to require HO-1-induction, which is more pronounced in a microenvironment resembling fibrotic liver tissue. This implicates that certain statins might be especially useful to support HCV therapy of patients at advanced stages of liver disease.

## Introduction

About 3% of the world's population are chronically infected with HCV [1], representing a cause of cirrhosis, steatosis and subsequent development of hepatocellular carcinoma (HCC) [2]. While for a long time a combinational therapy of interferon and ribavirin has been the best option for treatment of chronic HCV infection, sustained virologic response has now been markedly improved by treatment with HCV protease inhibitors in combination with pegylated interferon (IFN) alpha 2 [3]. Since evidence for escape mutants increases [4], alternative strategies, e.g. HMG-CoA-reductase inhibitors (statins), might be useful to support therapy.

Statins are among the most frequently prescribed medication worldwide. They interfere with endogenous cholesterol biosynthesis by competitively inhibiting its key enzyme HMG-CoA-reductase. Apart from this, statins have been described to possess other biological activities, e.g. exerting anti-proliferative effects in cancer [5]. Here mechanisms involving reduced availability of cholesterol biosynthesis intermediate products mevalonate and geranyl-geranyl pyrophosphate, but not a lack of cholesterol itself, seem to be play an important role [6], pointing to an inhibition of prenylation of small G-proteins and the Rho/ROCK pathway as crucial events in the anti-tumor activity of statins [7]. With respect to viral infections, statins have been shown to interfere with HIV replication and release *in vitro* [8], [9], while a combination of statins and caffeine inhibited influenza virus infection in mice [10]. With regard to HCV infection in patients, benefits of statin medication as well as the absence of effects have been described. Here clinical studies report that e.g. fluvastatin monotherapy reduced HCV replication [11] and enhanced efficacy of IFN alpha [12], while others could not confirm this observation [13], [14] or describe a rapid decrease of the viral load without significant effects on sustained virologic response [15]. The reasons for such inconsistent effects of statins on HCV replication are not clear, since the lack of prenylation, defined as the mechanism of anti-viral statin effects so far [16], should be achieved by all statins and there are no reports describing e.g. genetic polymorphisms being associated with hypo-responsiveness towards the cholesterol-lowering capacity of statins. In order to evaluate the usefulness of statins in HCV therapy we aimed to identify a reason for the variation of anti-viral activity, which might give explanation for the different anti-viral effects described in clinical studies.

It has been reported that statins are able to induce expression of the heme degrading enzyme heme oxygenase 1 (HO-1; Hsp32) in endothelial cells [17], smooth muscle cells [18], and macrophages [19]. We and others recently described that induction or over-expression of heme oxygenase 1 (HO-1) significantly interferes with HCV replication *in vitro* [20]-[21]. This effect could be attributed to the heme degradation product biliverdin [22] which increases endogenous anti-viral interferon signaling [22] and seems to directly inhibit the HCV protease NS3/4A [23].

In consequence we compared heme oxygenase 1 (HO-1)-induction and anti-viral activity of different statins like fluva- (FLV), simva- (SMV), rosuva- (ROV), atorva- (ATV) and pravastatin (PRV) *in vitro*. We found that all statins, except PRV, were able to induce HO-1 expression and reduce HCV replication. Anti-virally active statins interfered with the expression of the HO-1 transcriptional inhibitor Bach1 and induced expression of nuclear factor (erythroid-derived 2)-like 2 (NRF2) and the NRF2 co-factor Krueppel-like factor 2 (KLF2), which turned out to be crucial for HO-1-induction and anti-viral effects of statins. Induction of HO-1 by statins was sufficient to initiate interferon response *in vitro* and to support anti-viral effects of exogenous interferon or telaprevir incubation.

Interestingly, statin-mediated effects were more pronounced when cells were growing in an environment mimicking conditions found during advanced stages of liver disease.

## Materials and Methods

### Reagents

HMG-CoA-reductase inhibitors fluva- (FLV), simva- (SMV), prava- (PRV) (all: Cayman Chemical, Ann Arbour MI, USA), atorva- (ATV) (Sortis, Pfizer Pharma GmbH, Berlin, Germany) and rosuvastatin (ROV) (Crestor, Astra Zeneca, Wedel, Germany) as well as NS3/4A protease Inhibitor telaprevir (Janssen-Cilag Pharma GmbH, Wien, Austria) were dissolved in DMSO (Sigma Aldrich GmbH, Steinheim, Germany). As a vehicle control, DMSO was diluted to the concentrations used on statin-incubated cells. The Rho-kinase inhibitor Hydroxyfasudil (HA1100) (Tocris Bioscience, Bristol, UK) was dissolved in sterile water. Recombinant interferon alpha-2b (Intron A) was purchased from Essex Pharma, München, Germany.

### Cell culture and transfection

The replicon cell lines Huh-5-15 [24] and LucUbiNeo-ET [25] as well as their parental cell line Huh-7 [25] were cultured as described previously [22]. Cell viability was measured by using (3-4, 5-Dimethylthiazol-2-yl)-2, 5-diphenyltetrazolium bromide (MTT; Sigma Aldrich GmbH, Steinheim, Germany) according to the manufacturer's instructions. Transfections were performed using Lipofectamine™ 2000 (Invitrogen GmbH, Karlsruhe, Germany) according to the manufacturer's instructions. SiRNA target sequences: siKLF2: CTG CGG CAA GAC CTA CAC CAA (Qiagen GmbH, Hilden, Germany); siControl (GFP: AAT CTC AGG GTT CCT GGT TAA; Eurogentec Deutschland GmbH, Köln, Germany). ShRNA expressing vectors were based on the lentiviral pLKO.1 construct (RNAi Consortium vector collection [27]; and purchased from Sigma Aldrich GmbH (Steinheim, Germany). Target sequences for shRNA: HO-1: TGG GTC CTT ACA CTC AGC TTT CT; GFP: CAA CAA GAT GAA GAG CAC CAA [28]. Transfected cells were selected with puromycin (2 µg/mL). For virus production the plasmid pFK_I389_RLuc2ACore-3′-Jc1 harbouring the genome of a monocistronic reporter virus, referred to as JcR-2A, derived from the JC1 chimera [29] was used. Electroporation of Huh-7 cells and *in vitro* transcription of HCV RNA was performed as described previously [30].

### Infection with virus particles

Huh-7.5 cells were seeded into 12-well plates at 2×10^5^ cells/well 24 h prior to infection. Cells were infected with the HCV genotype 2a strain JC1 at an MOI of 0.5. After 2 h cells were washed 3 times with PBS, infection medium was changed and cells were incubated as indicated.

### Luciferase assay

Luciferase activity of LucUbiNeo-ET replicon cells was measured using the Luciferase Assay System (Promega, Mannheim, Germany), and normalized to the protein content of the individual sample.

### Immunofluorescence

To visualize HCV infection, E2 proteins were stained. Antibodies: human monoclonal A3R3 against E2 (a kind gift of Mansun Law, The Scripps Research Institute, La Jolla, CA, USA), chicken anti-human Alexa-488 (Molecular Probes, Life Technologies GmbH). The procedure included fixation (4% PFA; 20 min. at RT), permeabilization (0.1% Triton ×100; 4°C for 10 min) and blocking (5% BSA; 20 min. at RT). Pictures were taken using an inverted microscope (CKX41; Olympus, Hamburg, Germany) with an LCachN/20X/0.40 Phc/1/FN22 UIS objective.

### Detection of mRNA by RT-qPCR

RT-qPCR was performed as described previously [22], and on the ViiA™ 7 System (Life Technologies GmbH, Darmstadt, Germany), using TaqMan probes: HO-1 (Hs01110250_m1), GAPDH (Hs99999905_m1) and HCV (Pa03453408_s1). Oligonucleotides were obtained from Metabion International AG (Martinsried, Germany) and are summarized in Table 1.

**Table 1:** Oligonucleotide sequences for RT-qPCR.

Oligonucleotides	Sequences 5′-3′
5′BACH1	5′-GCAGATTGCCCACTTTCATT-3′
3′BACH1	5′-AGAGGTGGCTGTGGACATCT-3′
5′GAPDH	5′-TGATGACATCAAGAAGGTGG-3′
3′GAPDH	5′-CGACCACTTTGTCAAGCTC-3′
5′HO-1	5′-CCTGCTCAACATCCAGCTC-3′
3′HO-1	5′-CTACAGCAACTGTCGCCAC-3′
5′IFNalpha 2	5′-GCAAGTCAAGCTGCTCTGTG-3′
3′IFNalpha 2	5′-GATGGTTTCAGCCTTTTGGA-3′
5′IFNalpha 17	5′-AGGAGTTTGATGGCAACCAG-3′
3′IFNalpha 17	5′-CATCAGGGGAGTCTCTTCCA-3′
5′KLF2	5′-CACCAAGAGTTCGCATCTGA-3′
3′KLF2	5′-ACAGATGGCACTGGAATGG-3′
5'LDLR	5′-GTGCTCCTCGTCTTCCTTTG-3′
3'LDLR	5′-TAGCTGTAGCCGTCCTGGTT-3′
5′OAS 1	5′-CAAGCTCAAGAGCCTCATCC-3′
3′OAS 1	5′-TGGGCTGTGTTGAAATGTGT-3′
5′OAS 2	5′-ACAGCTGAAAGCCTTTTGGA-3′
3′OAS 2	5′-GCATTAAAGGCAGGAAGCAC-3′

### Western Blot

50 µg of total protein were fractionated by 12% SDS-polyacrylamide gel electrophoresis and blotted onto nitrocellulose membranes as described previously [22]. Antibodies: rabbit anti-HO-1 (1∶1000; Stressgen Biomol, Hamburg, Germany), mouse anti-NS4B (1∶1000; Abcam, Cambridge, UK), and mouse anti-GAPDH (1∶5000; HyTest Ltd., Turku, Finland).

### Preparation of Polyacrylamide Gel Supports

Polyacrylamide (PAA) gel supports of various elasticity were prepared on glass cover slips as described previously [31], [32], using modifications to the method initially described by Pelham and Wang [33].

### Statistical analysis

Results were analyzed using Student's *t* test, if two groups were compared and 1way ANOVA in combination with Bonferroni's Multiple Comparison Test if more than 2 groups were compared. All data are expressed as a mean ± SEM. * p≤0.05; ** p≤0.01; *** p≤0.001.

## Results

### FLV, SMV, ROV and ATV, but not PRV, interfere with HCV replication and induce HO-1

**Figure 1:**
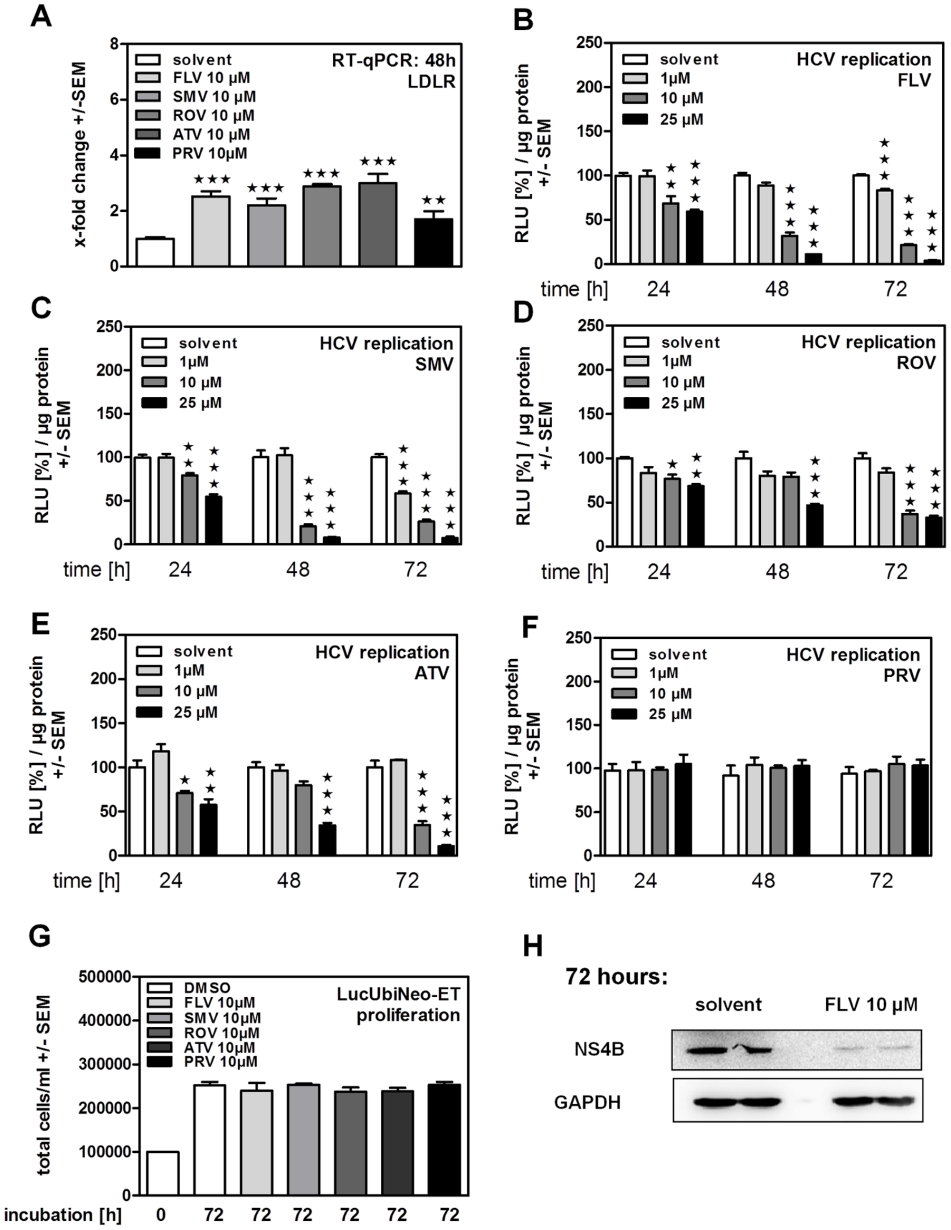
Inhibition of HCV replication by statins in genotype 1b replicon cells. LucUbiNeo-ET replicon cells were incubated in the presence of fluvastatin (FLV), pravastatin (PRV), simvastatin (SMV), rosuvastatin (ROV) or atorvastatin (ATV) at the indicated concentrations for 24, 48 or 72 hours. (**A**) Expression of the LDL-receptor (LDLR) was measured by real time RT-qPCR after 48 h of statin incubation (**B–F**) Dose- and time-dependent inhibition of HCV replication caused by statins was measured by luciferase reporter assay. Statin mediated effects on cell proliferation were measured after 72 hours of incubation (**G**) and confirmed by Western Blot analysis (**H**).

**Figure 2:**
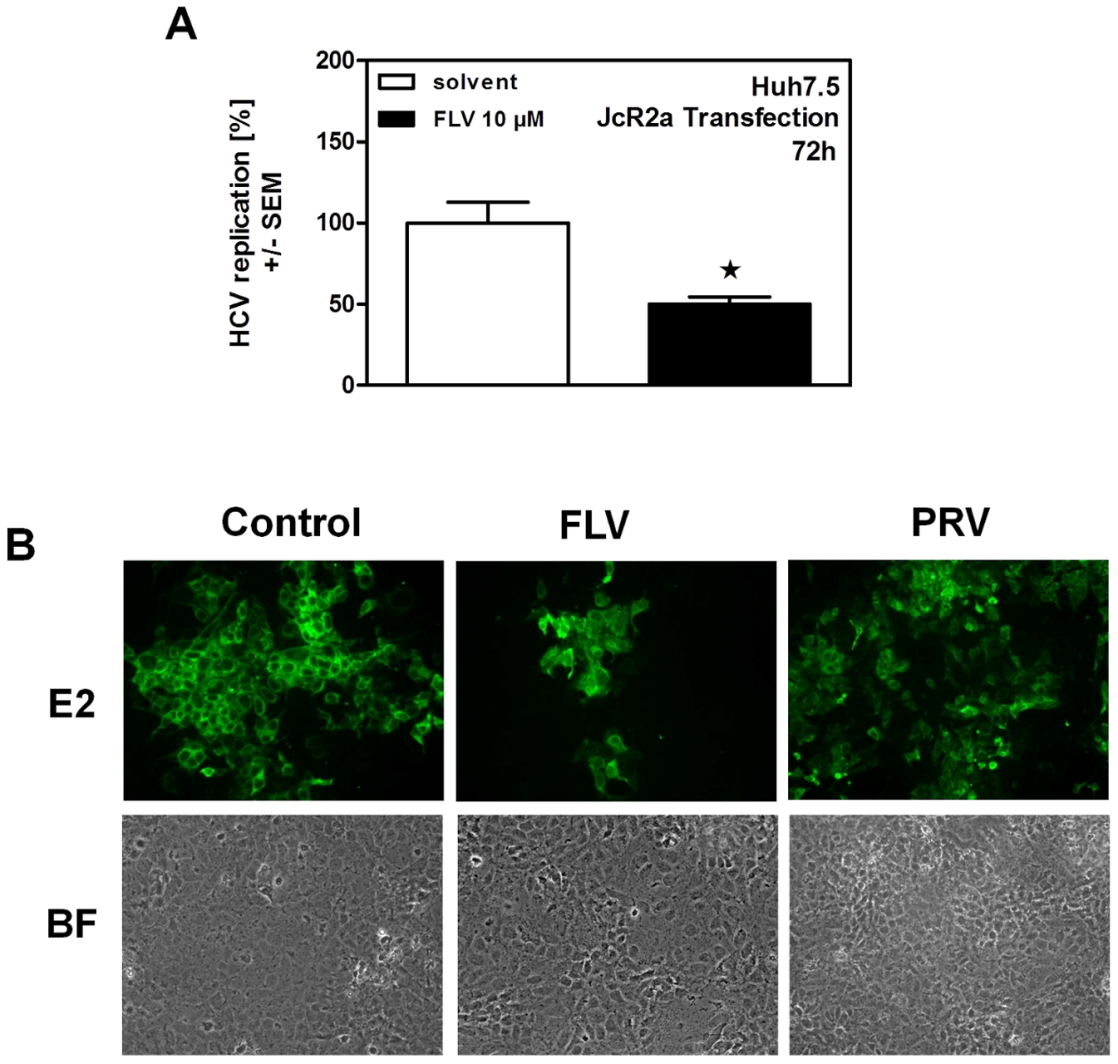
Inhibition of HCV in the infectious genotype 2a system. Huh-7.5 cells were transfected with the full length HCV clone JcR2a. HCV replication was measured by luciferase reporter assay after 72 hours of FLV incubation (**A**). Huh-7.5 cells were infected with the HCV genotype 2a strain JC1 and incubated with FLV or PRV for 24 hours. Viral replication was visualized by immunofluorescent staining of the HCV protein E2 (**B, upper pannel**). Cell viability was verified by bright field (BF) microscopy (**B, lower pannel**). Representative images pairs are shown.

Statins are widely used drugs to control biosynthesis of cholesterol and to reduce amounts of LDL-cholesterol by inducing LDL-receptor (LDLR) expression. Therefore, biological activity of statins used in our experiments was verified by measuring their ability to increase LDL-receptor (LDLR) expression by RT-qPCR (Fig. 1A). Investigating statin effects on HCV replication we found that fluva- (FLV) (Fig. 1B), simva- (SMV) (Fig. 1C), rosuva- (ROV) (Fig. 1D), and atorvastatin (ATV) (Fig. 1E) reduced HCV replication in the subgenomic replicon system in a dose- and time-dependent manner. On the other hand, PRV, while perfectly dissolved, was not able to reduce HCV replication (Fig. 1F). Anti-proliferative properties of statins which might interfere with HCV replication at [10 µM] were excluded by cell counting after 72 h of incubation (Fig. 1G). Results obtained by luciferase reporter assays were verified by Western Blot. As an example, Fig. 1H shows reduced HCV-non-structural protein NS4B expression after 72 hours of incubation with FLV. For further experiments we chose FLV as an anti-virally active and PRV as none anti-virally active representatives. We compared results obtained in the subgenomic replicon system (Fig. 1B, F, H) to the infectious HCV cell culture system. Here FLV reduced replication of the full-length HCV clone JcR2A, as shown by luciferase reporter assay (Fig. 2A) and staining of JC1-infected cells for the HCV structural protein E2 (Fig. 2B, upper center panel). As observed in the subgenomic replicon system (Fig. 1F) PRV, in contrast to FLV, did not reduce HCV replication and infection (Fig. 2B, upper right panel). Cell viability was verified by bright field microscopy (Fig. 2B, lower panel).

### Statin-induced HO-1 expression contributes to inhibition of HCV replication

**Figure 3:**
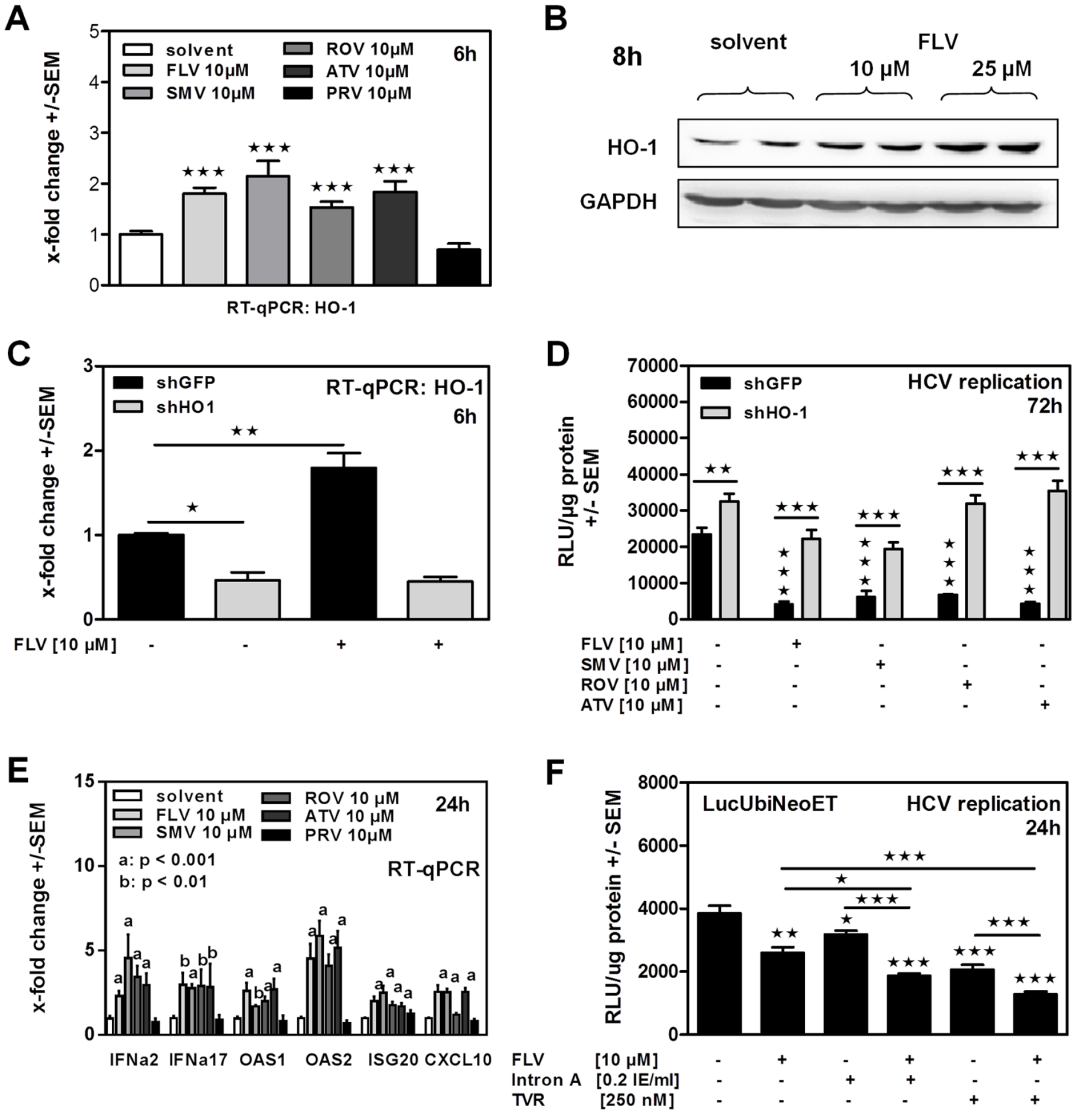
Statin-induced HO-1 expression contributes to inhibition of HCV replication and endogenous interferon response. HO-1-induction was measured by RT-qPCR in Huh-5-15 replicon cells after 6 hours of statin incubation (**A**). Western Blot analysis was used to visualize the HO-1 protein level was after 8 hours of incubation using 10 or 25 microM of FLV (**B**). LucUbiNeo-ET replicon cells stably expressing shRNA against HO-1 (shHO1) or a control gene (shGFP) were used to detect baseline or FLV-induced expression of HO-1 by RT-qPCR after 6 hours of incubation (**C**). ShGFP or shHO1 cells were incubated with statin for 72 hours. HCV replication was measured by luciferase reporter assay (**D**). LucUbiNeo-ET replicon cells were incubated in the presence of 10 microM of statins for 24 hours. Gene expression levels (interferon alpha 2; interferon alpha 17; interferon response genes) were analyzed by RT-qPCR (**E**). LucUbiNeo-ET cells were incubated with FLV, IFN alpha (Intron A) and telaprevir (TVR) alone or in combination for 24 hours. Single and combined effects on HCV replication were detected by luciferase assay (**F**).

Further investigations revealed that all statins, except PRV, significantly induced expression of the anti-viral enzyme HO-1 within 6–8 hours of incubation, shown by RT-qPCR (Fig. 3A) and Western Blot (Fig. 3B).

To investigate the contribution of HO-1-induction to the anti-viral effects of statins, we established replicon cell lines with a stable knockdown of HO-1 (shHO1) or a control gene (shGFP). In the shHO-1 cell line we observed an about 50% decrease of the HO-1 background expression, compared to the control cell line containing shGFP, while FLV was not able to increase HO-1 expression in those cells (Fig. 3C). Further investigations showed that a knockdown of endogenous HO-1 resulted insignificantly higher HCV replication in LucUbiNeoET cells (Fig. 3D) and interfered with statin-mediated inhibition of HCV replication (Fig. 3D). In a previous study, we could show that anti-viral effects of HO-1 are mediated by its degradation product biliverdin and that biliverdin is able to induce endogenous interferon response [22]. Our results show that all statins, except PRV, were able to induce endogenous interferon response (Fig. 3E). The combination of statin-incubation with interferon- or telaprevir -incubation significantly increased anti-viral effects induced by interferon or telaprevir alone (Fig. 3F).

### Statins induce HO-1 expression by a Bach1- and KLF2-dependent mechanism

**Figure 4:**
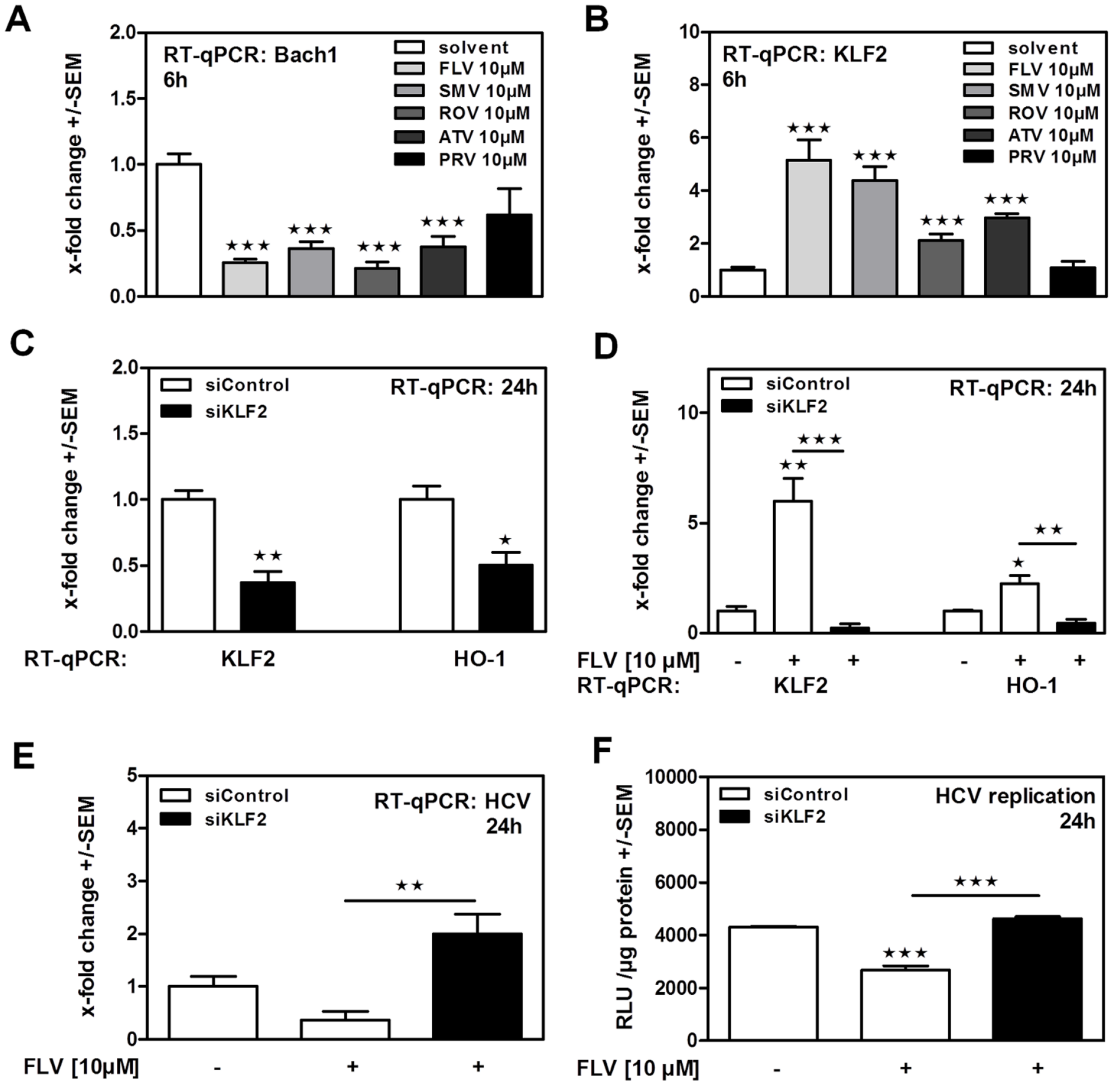
Statins promote HO-1 expression by reducing Bach1- and inducing KLF2- expression. Bach1 (**A**) and KLF2 (**B**) expression levels were measured in Huh-5-15 cells by real time RT-qPCR after 6 hours of statin incubation. LucUbiNeo-ET replicon cells were transfected with siRNA directed against KLF2 (siKLF2) or against a control gene (GFP; siControl) at [10 nM] (**C, D, E, F**). 24 hours after transfection expression of KLF2 and HO-1 was measured by RT-qPCR (**C**). Cells transfected with siRNA for 24 hours were further incubated with FLV for 24 hours. Expression of KLF2, HO-1 (**D**) **and HCV** (**E**) was measured by RT-qPCR, HCV replication was measured by luciferase reporter assay (**F**).

Investigating the mechanism of HO-1-induction by statins, we detected significantly reduced expression of the HO-1 transcriptional repressor Bach1 in statin-incubated cells (Fig. 4A). Here PRV incubation did not significantly reduce Bach1 expression, but showed a slight tendency (Fig. 4A). Expression levels of Krueppel-like factor 2 (KLF2), a cofactor of Nuclear factor (erythroid-derived 2)-like 2 (NRF2), which is involved in HO-1-induction [34], were found to be increased by all statins except PRV (Fig. 4B). Expression levels of Nuclear factor (erythroid-derived 2)-like 2 (NRF2) itself, or other factors involved in HO-1- induction, like Kelch-like ECH-associated protein 1 (KEAP1) or Hypoxia-inducible factor 1 (HIF1) alpha, were not increased by statin incubation (data not shown). Again using FLV as a model statin, we found that a knockdown of Krueppel-like factor 2 (KLF2) by transfection of siRNA (siKLF2) significantly reduced endogenous (Fig. 4C) as well as statin-induced (Fig. 4E) KLF2- and HO-1 expression. In consequence, a knockdown of KLF2 showed abrogated anti-viral effects of FLV as measured by luciferase reporter assay (Fig. 4E) or RT-qPCR (Fig. 4F).

### Matrix stiffness as a requirement for antiviral activity and HO-1-induction by statins

**Figure 5:**
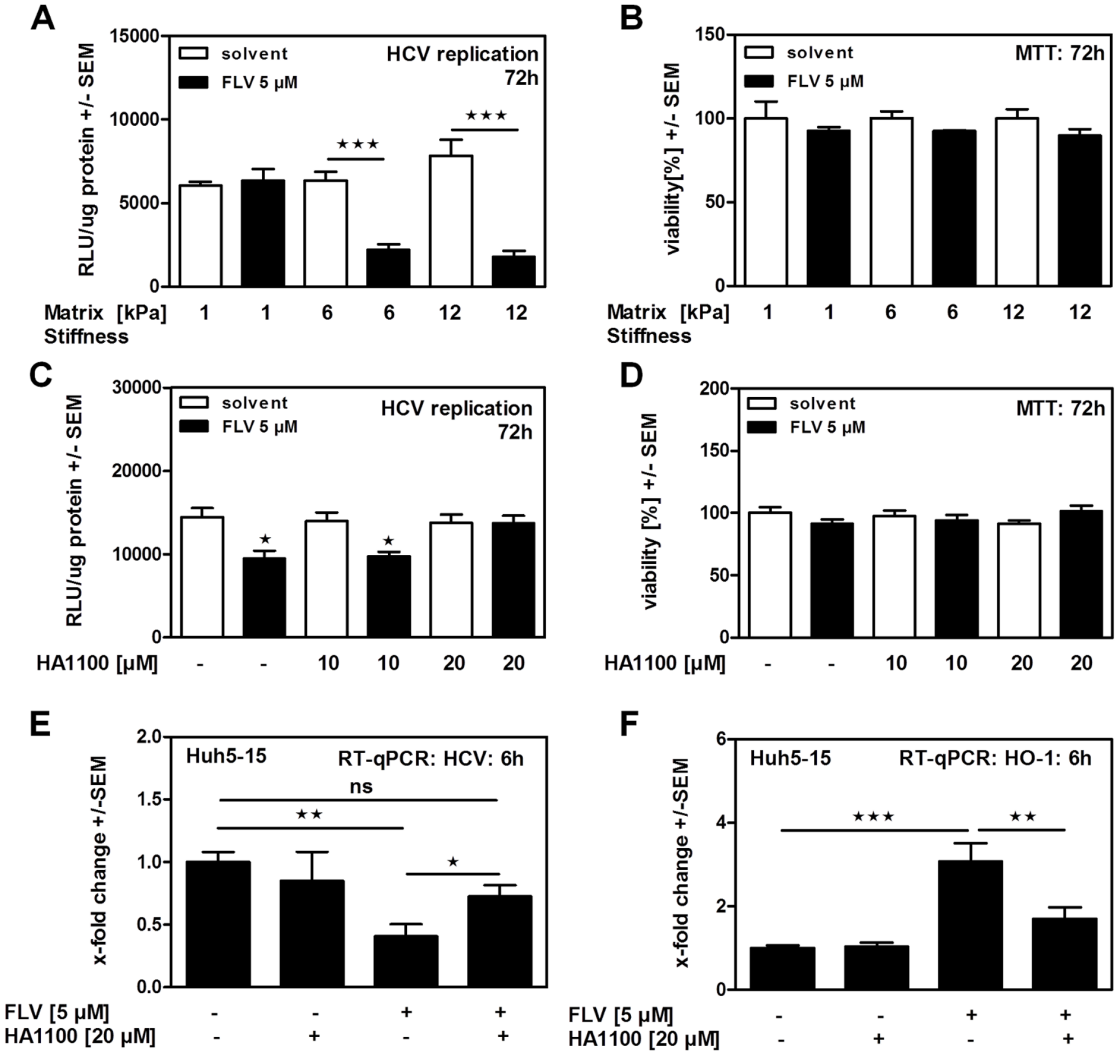
Matrix conditions predict anti-viral properties of statins. LucUbiNeo-ET replicon cells growing on PAA gel supports (1–12 kPa: 1 kPa = soft; 12 kPa = stiff) were incubated with FLV for 72 hours. Effects on HCV replication were measured by luciferase assay (**A**). For the same settings cell viability was measured by MTT assay (**B**). LucUbiNeo-ET replicon cells were incubated with increasing concentrations of the Rho kinase inhibitor HA1100 alone or in combination with FLV at 5 µM for 72 hours. HCV replication was measured by luciferase reporter assay (**C**). Viability of LucUbiNeo-ET replicon cells was monitored for the same settings by MTT assay (**D**). Huh-5-15 replicon cells were incubated with FLV with or without coincubation with HA1100 for 6 hours. HCV replication (**E**) as well as HO-1 expression (**F**) was measured by RT-qPCR.

Viral infections of the liver frequently result in chronic inflammation and formation of fibrosis. In order to investigate how these conditions might influence anti-viral effects of statins we prepared Polyacrylamid (PAA) gels of different stiffness as supports for the replicon cell culture system, mimicking physiological (soft) or fibrotic (stiff) liver tissue. In fact, hepatocellular carcinoma (HCC) cell morphology as well as proliferation has been shown to be stiffness-dependent [31]. Our results show that the anti-viral activity of FLV was decreased when replicon cells were growing on a soft matrix (Fig. 5A), while there were no adverse effects on cell viability (Fig. 5B). In a second approach we used the Rho kinase inhibitor HA1100 to mimic decreased environmental stiffness by interfering with the matrix stiffness sensing of these cells. In this environment anti-viral effects of statins were also decreased, as measured by luciferase reporter assay (Fig. 5C) and RT-qPCR for viral replication (Fig. 5E), while cell viability was not affected (Fig. 5D). Furthermore, incubation of cells with the Rho kinase inhibitor reduced the ability of FLV to induce HO-1 (Fig. 5F).

## Discussion

With increasing average body mass in western countries and the increased risk of developing cardiovascular diseases, HMG-CoA-reductase inhibitors have found their place among the most frequently prescribed medication. Although the primary indication for statin prescription is treatment of hypercholesteremia to prevent adjacent secondary events, e.g. coronary problems, evidence is increasing that statins might also display beneficial effects on other kinds of diseases [5], [8], [10], [11]. It has recently been shown that statins, except PRV, enhance effects of HCV-targeted therapy by NS3- or NS5-inhibitors *in vitro*, and might reduce resistance development [35]. This is in line with our results showing that a combination of FLV and telaprevir was significantly more effective in reducing HCV replication, compared to effects of each compound alone. In addition we observed that FLV significantly increased anti-viral effects of interferon, giving rise to the speculation that statin supplementation might help reducing therapeutic levels of interferon and its adverse side effects in patients.

These results are promising but do not generally reflect anti-viral statin effects in patients [36], [37], [38]. Clinical studies reported that FLV monotherapy reduced replication of HCV [11] and enhances efficacy of interferon alpha [39], though others could not confirm that observation [40], [41] or found a rapid decrease in viral load without significant effects on SVR [15]. Knowledge of mechanisms involved in anti-viral effects of statins might help to understand under which circumstances statins might support antiviral therapy. To date, inhibition of prenylation has been suggested as the mechanisms behind anti-viral activity of statins [42]. In the process of cholesterol biosynthesis inhibition, statins reduce generation of the isoprenoids, which are necessary for post-translational modifications and activation of cellular proteins [43], thereby regulating cell survival and cell growth [44]. It has to be taken into account that subgenomic replicon system are based on the fast proliferating human hepatoma cell line Huh7 and that cellular integrity is a prerequisite for cell proliferation and efficient HCV replication. At higher concentrations or longer statin incubation time we in fact observed a loss of cell viability in human and mouse hepatoma cell lines which was reverted by restoration of prenylation [6]. This might disguise more specific mechanisms. Moreover, inhibition of prenylation is a general mechanism of statin action and does not provide an explanation why e.g. PRV does not interfere with HCV replication.

Statins have been shown to regulate gene expression. Deficiency in cholesterol leads to the activation of sterol regulatory element-binding proteins (SREBPs), transcription factors, which increase expression of LDL-receptor (LDLR), subsequently leading to reduction of LDL-cholesterol [45]. Our results show that statins were able to induce expression of the anti-viral enzyme HO-1 in replicon-containing cells by reducing expression of Bach1, a transcriptional repressor for HO-1 [46], as well as by activation of NRF2 due to increased availability of its cofactor KLF2. KLF2 in fact seems to be the key factor since its knockdown reduced HO-1 expression and restored HCV replication in the presence of statins. It has recently been shown that HO-1 interferes with HCV replication [20]–[22] via its product biliverdin [22], [23]. Moreover, we could show that statins were not able to reduce HCV replication in cell lines carrying a stabile knockdown of HO-1 or KLF2. Our results also show that PRV was not able to induce HO-1- or KLF2-expression in replicon cells, providing a possible explanation for missing effects of PRV on HCV replication. Taken together our results indicate that HO-1-induction might be a key event in anti-viral activity of statins.

Aiming to provide a possible explanation on inconsistent anti-viral effects of e.g. FLV in patients we investigated the role of matrix stiffness, corresponding to degrees of cirrhosis. Matrix stiffness has so far been mostly neglected in the study of therapeutic responses in cell culture. Since hepatocytes under physiological conditions reside in a soft environment *in vivo*, the regular culture on plastic surfaces has to be considered a non-physiological stiff environment, resembling fibrotic or cirrhotic conditions. It has already been shown that environmental stiffness affects interferon treatment and thereby therapeutic responses in HCV infection [47]. Here we provide evidence, that matrix stiffness modulates the response of HCV replicon cells towards therapeutically used statins. In contrast to observations regarding interferon, we found increased effects of fluvastatin on HCV replication in replicon cells cultured on stiff matrices as well as cell culture plastic material, while effects on HCV replication as well as HO-1 expression were largely diminished under soft matrix conditions. Although these results might suggest a benefit of statin use in adjunct to anti-viral therapy in patients with advanced liver disease, further studies in animal models and observational studies in humans are needed to prove this speculation. While statins have so far been used cautiously in patients with advanced liver diseases, new data suggest, that they might be safely used even in patients with cirrhosis [48].

As an additional benefit we observed that statins were able to induce endogenous interferon response under cell culture conditions which are comparable cirrhotic tissue conditions. Since exogenous interferon treatment has been shown to display severe side effects, stimulation of endogenous interferon response might be an alternative attempt or at least help to reduce therapeutic doses of exogenous interferon. Hence, FLV might further support therapy especially under stiff matrix conditions by inducing endogenous interferon response. The direct antiviral acting (DAA) compound telaprevir is metabolized in the liver by cytochrome P-450 (CYP) isoenzyme 3A4 to the inactive (α-ketoamide bond) metabolites and the active *R*-diastereomer. Telaprevir is both a substrate and an inhibitor of the isoenzyme [48]. Atorva-, lova- and simvastatin are metabolized by the CYP3A4 isoenzyme [49]. Therefore, a combination of telaprevir with these statins would decrease their metabolism leading to increased blood concentrations, with a potential risk of detrimental side effects. On the other hand, FLV is metabolized by the CYP2C9 isoenzyme [50], which is not known to be affected by telaprevir. Therefore the combination of telaprevir and FLV might be beneficial. To verify this hypothesis further studies are necessary.

Taken together our data provide evidence that HO-1-inducing statins might represent a beneficial therapeutic support for HCV patients with advanced liver disease.
